# Surgical Technique in Distal Pancreatectomy: A Systematic Review of Randomized Trials

**DOI:** 10.1155/2014/482906

**Published:** 2014-05-29

**Authors:** Filip Čečka, Bohumil Jon, Zdeněk Šubrt, Alexander Ferko

**Affiliations:** ^1^Department of Surgery, Faculty of Medicine and University Hospital Hradec Králové, Sokolská 581, 500 05 Hradec Králové, Czech Republic; ^2^Department of Field Surgery, Military Health Science Faculty Hradec Králové, Defence University Brno, Třebešská 1575, 500 01 Hradec Králové, Czech Republic

## Abstract

Despite recent improvements in surgical technique, the morbidity of distal pancreatectomy remains high, with pancreatic fistula being the most significant postoperative complication. A systematic review of randomized controlled trials (RCTs) dealing with surgical techniques in distal pancreatectomy was carried out to summarize up-to-date knowledge on this topic. The Cochrane Central Registry of Controlled Trials, Embase, Web of Science, and Pubmed were searched for relevant articles published from 1990 to December 2013. Ten RCTs were identified and included in the systematic review, with a total of 1286 patients being randomized (samples ranging from 41 to 450). The reviewers were in agreement for application of the eligibility criteria for study selection. It was not possible to carry out meta-analysis of these studies because of the heterogeneity of surgical techniques and approaches, such as varying methods of pancreas transection, reinforcement of the stump with seromuscular patch or pancreaticoenteric anastomosis, sealing with fibrin sealants and pancreatic stent placement. Management of the pancreatic remnant after distal pancreatectomy is still a matter of debate. The results of this systematic review are possibly biased by methodological problems in some of the included studies. New well designed and carefully conducted RCTs must be performed to establish the optimal strategy for pancreatic remnant management after distal pancreatectomy.

## 1. Introduction

Distal pancreatectomy is the surgical procedure of choice for the treatment of lesions in the body and tail of the pancreas. The mortality associated with this procedure has decreased rapidly in the past decades due to refinements in operative technique, introduction of new surgical devices, and improvements in postoperative care, including new interventional radiology techniques; however, morbidity remains high [1–6]. The main reason for postoperative morbidity is the postoperative pancreatic fistula (POPF), which is also regarded as the most ominous complication [7]. POPF is not a life-threatening condition in most cases, but nevertheless it prolongs the hospital stay, increases the cost of the treatment and delays adjuvant treatment in malignant disease [8].

Distal pancreatectomy is performed less frequently than pancreaticoduodenectomy [5, 9]. This is because of the lower incidence of pancreatic disease in the body and tail of the pancreas and the later appearance of clinical symptoms in this part of the organ. Pancreatic adenocarcinoma is found in the left part much less frequently than in the head. However, continuous improvement in the quality of imaging studies and frequent use of ultrasonography for all kinds of indications have resulted in higher incidence of the findings of lesions in this part of the pancreas, for example, asymptomatic cystic or endocrine tumors [9].

Compared to pancreaticoduodenectomy, fistulas that occur after distal resections are usually clinically less severe [5, 9]. Sauvanet et al. suggested that POPF originating from pancreaticoenteric anastomosis seems to have a worse prognosis than POPF originating from a pancreatic remnant [10]. This may be due to the activation of pancreatic juice by enterokinase, which is a necessary mechanism that stimulates the proteoclastic activity of various pancreatic enzymes [11]. This process may contribute to the differences between POPFs after operations that require enteric reconstructions (pancreaticoduodenectomy and central pancreatic resection) and those that do not (distal pancreatectomy and enucleation). Pratt et al. suggested that clinically relevant fistulas after pancreaticoduodenectomy require more aggressive management in intensive care settings compared to those that occur after distal resections. Surgical exploration, when indicated, is more often urgent. On the other hand, fistulas that occur after distal resections often require prolonged drainage of intra-abdominal collections and multiple hospital readmissions, usually for image-guided percutaneous drainage [5].

As POPF has significant clinical and economic consequences, attention has focused on lowering the POPF rate. Besides the use of somatostatin or its analogues in high-risk patients [12], these efforts comprise mainly surgical technique and the strategy for pancreatic remnant management. New methods have emerged including experimental studies [13] in order to develop new techniques in distal pancreatectomy.

There have been few retrospective studies to compare the various techniques for management of the pancreatic remnant. The results are heterogeneous and often contradictory: several authors have shown lower fistula rates in manual oversewn closure compared to stapler transection [6, 14–16], while others favored stapler [17–19]. Even though some of the new surgical techniques show promising results in a retrospective cohort setting [20], the expected advantage diminishes in randomized controlled trial (RCT) [21]. Another example would be the use of pancreatic duct stent with favorable results in a retrospective study [22, 23], but not confirmed in a randomized trial [24]. This shows the importance of well-designed RCTs in decision making and estimation of treatment effect in surgical interventions [25, 26].

Several reviews have studied the various surgical techniques in distal pancreatectomy. They focused mainly on the two most commonly practiced interventions: stapler versus manual oversewn closure of the pancreatic remnant [27–29]. However, there are more surgical techniques available and more issues to face. Other less common techniques include pancreatic transection using various energy devices, reinforcement of the stump with a seromuscular patch or pancreaticoenteric anastomosis, sealing with fibrin sealants, the use of various meshes, and pancreatic stent placement [28, 30].

Two meta-analyses which comprised mostly retrospective trials [28, 29] did not achieve firm conclusions. Zhou et al. showed a trend in favor of the stapler-closure technique, although it did not reach statistical significance [29]. The meta-analysis performed by Knaebel et al. favored the stapler closure as well; however, the result was not statistically significant [28]. Both authors concluded that a large RCT must be conducted in order to confirm the results of the meta-analyses. This was accomplished by Diener et al. in the DISPACT trial [27]. This again shows the importance of well-designed RCTs and their predominance over retrospective studies. For this reason we carried out a systematic review of RCTs dealing with surgical techniques in distal pancreatectomy to summarize up-to-date knowledge on this topic.

## 2. Methods

### 2.1. Search Strategy and Study Selection

We searched the Cochrane Central Registry of Controlled Trials, Embase, Web of Science, and Pubmed (=Medline) for relevant articles published from January 1990 to December 2013. The search was performed independently by two authors (FC and BJ) using the terms: “distal pancreatectomy,” “pancreatic resection,” “pancreatic fistula,” “pancreas,” and “postoperative complication.” The full search strategy is shown in the appendix (Literature search).

The reference lists of relevant studies were screened to retrieve any further potential studies. No unpublished data or data from abstracts were encountered or used. No language restriction was applied to the search.

Abstracts of all potentially relevant articles were read and assessed. All studies comparing various strategies in distal pancreatectomy were retrieved, and only randomized clinical trials were included in the systematic review.

### 2.2. Inclusion and Exclusion Criteria

We considered only RCTs comparing various strategies and surgical techniques of distal pancreatectomy for the review. Nonrandomized trials and clinical observational studies were excluded. Studies without data available for retrieval or studies describing only one technique were excluded. Studies comparing various techniques in pancreaticoduodenectomy or other procedures were also excluded, as were experimental studies on animals.

### 2.3. Data Analysis and Statistical Methods

All data of selected studies were analyzed independently by two reviewers (FC and BJ). We extracted data on methodology, level of evidence, population, interventions, outcome measures including POPF rate, postoperative morbidity and mortality, and definition of pancreatic fistula [31, 32]. Disagreements were resolved in group discussions. Methodology followed the standard guidelines outlined in the Cochrane Handbook for Systematic Reviews of Interventions [33] and the PRISMA statement (Preferred Reporting Items for Systematic Reviews and Meta-Analyses) [34]. The risk of bias of the studies was assessed independently by two authors based on individual components.

## 3. Results

**Figure 1:**
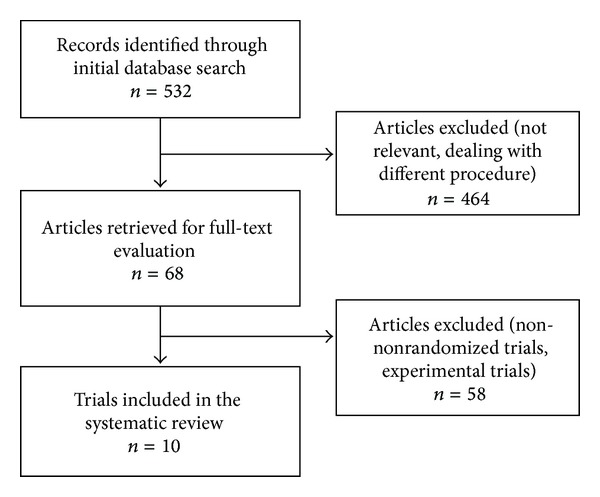
Flowchart of the literature search strategy.

The initial search strategy retrieved 532 publications. 464 were excluded in the primary selection (not relevant or dealing with pancreaticoduodenectomy or another procedure) and 58 were excluded in the secondary selection after reading the full-text of the potentially relevant studies (nonrandomized trials, experimental trials). Ten RCTs were identified and included in the systematic review, with a total 1286 patients being randomized (samples ranging from 41 to 450) [21, 24, 27, 35–41]. The reviewers came to agreement for application of the eligibility criteria for study selection. A flowchart of the literature search strategy is shown in Figure 1.

The main characteristics of the selected trials are shown in Table 1 [21, 24, 27, 35–41]. Three studies were multicentric [27, 38, 41], one study was from 2 centers [21], and the others represent single-center experience [24, 35–37, 39, 40]. The definition of POPF was not uniform throughout the studies, and hence the POPF rate cannot be compared among the studies. Not surprisingly, the POPF rate ranged from 3.7% up to 68.5%. It was clearly shown that POPF definition is the most important factor of the POPF rate [31].

Only two RCTs compared stapler versus hand-sewn closure [27, 37]. Meta-analysis of those two techniques was included in the report of the DISPACT trial [27]; it did not show a difference between the two techniques (odds ratio OR 0.87; 95% CI 0.3–2.55; *P* = 0.80). Whereas the results of 16 observational studies were in favor of stapler closure (OR 0.68; 95% CI 0.51–0.89; *P* = 0.006); it emphasizes the limitations of nonrandomized trials in surgery again [27]. We found it unnecessary to perform the same meta-analysis again. Comparability between the other studies was compromised because of the heterogeneous surgical techniques and approaches, such as the various methods of pancreas transection, reinforcement of the stump with a seromuscular patch or pancreaticoenteric anastomosis, sealing with fibrin sealants, and pancreatic stent placement. It was thus not possible to conduct a meta-analysis of such trials. The following studies were identified and analyzed.

Suzuki et al. reported the results of the first RCT comparing the application of fibrin glue with a control group [35]. This small RCT contained 56 patients; fibrin glue was applied in 26 patients to the suture line on the proximal stump, with ligation of the main pancreatic duct. In the control group, the transection and suture were carried out in the same manner, only without fibrin glue application. POPF occurred less frequently in the fibrin glue group compared to the control group. The validity of the results must be questioned for several reasons: firstly, the small sample size; secondly, there was poor selection of the study population, 75% of patients had been operated on for gastric cancer; and thirdly, the data on postoperative morbidity was not shown.

The second study was also conducted by Suzuki et al. [36]. The authors reported the value of ultrasonic dissection in a RCT containing 58 patients. In the experimental group, the pancreas was transected by ultrasonic dissector, and even small pancreatic ducts were exposed and ligated. POPF occurred less frequently in the experimental group compared to the control group. The drawback of the new technique is the need to ligate all the pancreatic ducts; approximately 20–30 tubes including the pancreatic ducts and small blood vessels were ligated per patient, resulting in longer time required for division of the pancreas in the ultrasonic dissection group (23.0 minutes versus 9.1 minutes, resp.; *P* = 0.039). Moreover this trial was subject to the same drawbacks lowering its credibility as the trial mentioned above, that is, the small sample size, 86% of the patients operated on for gastric cancer, and data on morbidity and mortality not shown. Furthermore, the authors did not explain why they did not use fibrin glue, after having shown in their previous study that it produced superior results.

Bassi et al. conducted a pilot RCT with 69 patients being randomized into 5 groups which included suture closure, suture closure ± fibrin glue, suture closure ± polypropylene mesh, pancreaticojejunostomy, and stapler closure [37]. Although the POPF rates ranged from 7.1% to 33.3%, the results were not statistically significant. This is clearly due to the small sample size of this pilot study. However, the authors showed interesting comparison of various techniques of management of the pancreatic stump.

Suc et al. conducted a multicenter RCT including 182 patients from 15 centers, of whom 41 underwent distal pancreatectomy [38]. A wide range of techniques was allowed, such as suture-closure, Roux-en-Y jejunal loop anastomosis, and omentoplasty. Patients were randomized to receive temporary fibrin glue occlusion of the main pancreatic duct or not. The pancreatic fistula rate did not vary between the groups. This study was underpowered because the sample size was calculated for both pancreaticoduodenectomy and distal pancreatectomy. Moreover, the heterogeneity of the surgical techniques and the multicenter nature of the study decreased the credibility of the results.

Oláh et al. conducted a RCT comparing stapler closure versus stapler closure with jejunal seromuscular patch [39]. Overall pancreas-related morbidity (POPF, intra-abdominal collection, or both) was significantly lower in the jejunal patch group; however, the incidence of clinically significant POPF grades B/C was comparable between the groups. The authors concluded that addition of a jejunal seromuscular patch to stapler closure reduced the rate of pancreatic fistula and abdominal collections, but it did not affect clinically relevant outcomes. This study was underpowered as the number of analyzed patients did not reach the calculated sample size.

A large multicenter trial was designed to compare suture closure versus stapler closure [27]. The trial was well designed and carefully conducted among 21 centers in Europe; 450 patients were randomized and 296 analyzed. The primary end-point was POPF and death until the 7th POD. The authors themselves admit that the assessment period for the POPF up to 7th POD might be too short. Both methods were shown to be comparable in terms of POPF rate, mortality, overall morbidity, and hospital stay. Unfortunately the authors did not analyze the cost of the treatment; opponents of the stapling method argue against the higher price of the device. The operating time with stapler was not significantly shorter; thus, use of the stapler does not speed up the procedure.

A small RCT by Frozanpor et al. showed no benefit for prophylactic pancreatic duct stenting [24]. Even though the study may be underpowered, there was not even a trend towards a lower rate of complications in the stented group. Moreover, according to the authors prophylactic stenting may even be harmful. One of the many contributing factors could be luminal bacteria seeding through the stent [42].

Hamilton et al. showed that mesh reinforcement decreases the rate of clinically significant POPF (grades B/C) and the overall POPF rate [40]. Unfortunately the authors did not use the generally accepted POPF definition of ISGPF, thus precluding comparison of the results with those from other studies. The pancreatic fistula definition was quite narrow, but nevertheless the POPF rate was still relatively high (47%). This was the only study in which the results indicated a credible advantage for one technique over another.

A multicenter RCT by Montorsi et al. showed that the application of a biological sealing agent (TachoSil) over the pancreatic stump as an addition to standard suturing or stapling did not result in a significant reduction in the overall POPF rate [41]. The amylase level in the drain fluid was lower in the TachoSil group on day 1, which suggests that TachoSil may be effective in sealing the pancreatic remnant in the immediate postoperative period. There was a certain degree of heterogeneity regarding the type of surgery (laparotomy versus laparoscopy, suture versus stapler, and spleen preservation or not). The heterogeneity was even greater because of the large number of centers involved (19). However, the authors claim that the results reflect real-life practice more conclusively.

Carter et al. sought to decrease the POPF rate by adding a falciform ligament patch and fibrin glue to the pancreatic remnant in a dual-institution randomized study [21]. However, they were not successful; the POPF rates were not significantly different between the groups at a scheduled midterm data analysis (at 52.5% enrolment). Thus the study was closed to enrolment. This study has several drawbacks; firstly, complications beyond 30 days postoperatively were not fully included; then the surgical technique used in the trial was not consistent, stapler or manual suture; and finally, the method of constructing the falciform ligament patch prevents a pure comparison with the literature.

## 4. Discussion

This systematic review includes 10 RCTs, which described the techniques used for pancreatic remnant management after distal pancreatectomy [21, 24, 27, 35–41].

The surgical techniques ranged from the standard techniques used most commonly (stapler closure and manual hand-sewn closure [27, 37]) to techniques used less frequently (ultrasonic dissection, closure with jejunal seromuscular patch, polypropylene mesh [36, 37, 39]). Fibrin glue was used in various ways: simple application of fibrin glue on the suture line [35, 37], fibrin glue occlusion of the main pancreatic duct [38], and falciform ligament patch with fibrin glue [21]. Other techniques included application of a biological sealing agent (TachoSil) [41], prophylactic pancreatic duct stenting [24], or pancreaticojejunostomy [37]. Because of the heterogeneous surgical techniques and approaches, the comparability between the studies was compromised.

Moreover, different definitions of morbidity and POPF were used. Only five of the included studies [21, 24, 27, 39, 41] used the definition according to ISGPF [32] which nowadays is the most commonly used and has been validated [8, 43].

Pancreatic fistula according to the ISGPF was defined as output via operatively or postoperatively placed drains of any measurable volume of drain fluid on or after postoperative day 3, with amylase content greater than three times the upper normal serum value. Three grades of pancreatic fistula were determined according to the clinical severity [32]. Grade A fistula, also called “transient fistula” has no clinical impact. It requires little or no change in the clinical management of the patient. Grade B fistulas are symptomatic and clinically apparent, and they require changes in clinical management or adjustment of the clinical pathway. The patients are usually supported by enteral or parenteral nutrition, and the peripancreatic drains are usually kept in place or new drains may be inserted. Grade C fistulas are severe and clinically significant, requiring major adjustments in clinical management. Clinical intervention is aggressive; patients are often in the intensive care unit (ICU) and have enteral or parenteral nutrition, antibiotics, and somatostatin analogues. Surgical revision may be indicated in some cases [32].

The other studies used different criteria such as amylase concentrations in the fistula fluid, fluid amounts, methods of detection, and time points for description. Not surprisingly, the POPF rates vary from 3.7% to 68.5%; thus, it is not possible to make comparisons between individual studies and surgical techniques. When the various definitions of POPF are applied to identical groups of patients, the rate of pancreatic fistula can range from 10% to 29% according to which definition is applied [31]. Naturally, broad POPF definition will result in higher POPF rates [31, 32].

RCT is the method showing the best evidence excluding possible bias which may be encountered in nonrandomized retrospective or cohort studies [26]. RCT is regarded as the gold standard for evaluating results of various surgical methods or other interventions. A well designed RCT guards against systematic and random errors. RCTs minimize the risk of confounding factors; they provide the highest level of evidence in terms of validity as they are more likely to closely reflect a true effect than other types of studies [26].

Even though some of the new surgical techniques show promising results in the retrospective cohort setting [20], the expected advantage diminishes in RCT [21]. Even results of meta-analyses including mostly retrospective or cohort studies [28, 29] are compromised compared to a well-designed and well-conducted RCT [27]. We decided to conduct a review solely of RCTs to gather together the up-to-date evidence in surgical techniques in distal pancreatectomy.

The results from RCT are more valid and trustworthy than nonrandomized retrospective or cohort studies, but only when it is well conducted. The results of small, underpowered, and poorly designed surgical RCTs with high risk of bias may be overvalued because their design provides them with unwarranted credibility [25].

RCTs in surgical trials should adhere to methodological principles to minimize errors. The methods include sequence generation (randomization), concealment of allocation, blinding, intention-to-treat principle, complete followup, and sample size calculation [33]. The included RCTs have their drawbacks (Table 2). Allocation concealment relates to what happens before randomization of the patients and seeks to eliminate selection bias. Blinding relates to what happens after randomization and seeks to reduce performance and detection bias [25]. Several individuals have potential to introduce bias if they have knowledge of which intervention the participants have received. Obviously surgeons cannot be usually blinded, but participants, nursing staff, data collectors, and outcome assessors can be blinded [25]. Most of the included trials misinformation are about concealment allocation and blinding.

The first three trials are missing important information about group size calculation, randomization, blinding, and followup [35–37] and thus were assessed as having a high risk of bias. The risk of bias of the study performed by Suc et al. is unclear; mainly because it was a part of a larger study including both pancreaticoduodenectomy and distal pancreatectomy [38].

Because of the limits of the included studies, we must analyze the results with caution. The studies by Carter et al., Frozanpor et al., Diener et al., Suc et al., and Montorsi et al. did not show significant differences between the study arms [21, 24, 27, 38, 41]. Oláh et al. found a lower rate of overall pancreas-related complications in the seromuscular patch group over the stapling alone group (11.4% versus 31.4%, *P* = 0.041), but there was no significant difference between the groups regarding the complications requiring intervention (5.7% versus 14.3%; *P* = 0.428) [39]. Such conflicting results might be due to the underpowered sample size.

The only study with low risk of bias which showed significant advantage of one method over another was conducted by Hamilton et al. [40]. The authors showed that mesh reinforcement to stapler suture reduces the rate of clinically significant POPF grades B/C (1.9% versus 23.9%; *P* = 0.001). However, the POPF rate was still on the high side despite the POPF definition used.

It is difficult to reach conclusive results and draw firm conclusions due to the drawbacks and possible bias of the included studies, various surgical techniques, and various POPF definitions. The best method of pancreatic remnant management in distal pancreatectomy is still debated. We can speculate that the perfection of a technique at each individual institution or by an individual surgeon is just as important as the actual technique applied.

New studies are currently underway which compare various surgical techniques of pancreatic remnant management after distal pancreatectomy (Table 3). An RCT comparing the radiofrequency ablation device (Tissuelink) technique with the stapling device (SEAMGUARD) is being conducted in the USA. Another Japanese multicenter RCT compares duct to mucosa pancreaticojejunal anastomosis and simple stapler closure. A Korean multicenter RCT compares TachoComb and polyethylene glycolic acid (PGA) in distal pancreatectomy. TachoComb is a ready-to-use hemostatic agent consisting of a collagen sheet coated on one side with human fibrinogen, bovine thrombin, and bovine aprotinin. Polyglycolide or Polyglycolic acid (PGA) is a biodegradable, thermoplastic polymer and the simplest linear, aliphatic polyester. Both arms will be compared to a control arm, in which no mesh will be applied to the cut surface of the pancreas. The German trial DISCOVER is testing the method of coverage of the pancreatic remnant with a falciform ligament [44]. Another study from the Massachusetts General Hospital, Boston, USA, was planned to lower the POPF rate with pancreatic duct stenting prior to the surgical procedure, the same technique which was used by Frozanpor et al. [24]. However, this study was withdrawn prior to enrolment due to the high risk of pancreatitis according to the authors.

## 5. Conclusion

Management of the pancreatic remnant after distal pancreatectomy is still a matter of debate. It remains a clinically significant problem. The results of this systematic review are possibly biased by methodological problems within some of the included studies. New well designed and carefully conducted RCTs must be performed to establish the optimal strategy for pancreatic remnant management after distal pancreatectomy. Such studies are currently underway and we can eagerly await their results.

## Figures and Tables

**Table 1:** The main characteristics and results of the selected trials.

Reference	Year		Age	Sex (M/F)	Definition of pancreatic fistula	Interventions	Group size	Mortality	Morbidity	Fistula rate	*P*
Suzuki et al. [35]	1995	SC	58,7 ± 15,5* 61,5 ± 9,4*	19/7 21/9	Any amount, concentration 3x normal serum value lasting for at least 7 days	Fibrin glue control group	26 30	1,8%	N/A N/A	15,4% 40,0%	0,04

Suzuki et al. [36]	1999	SC	57,7 ± 10,9* 58,5 ± 11,9*	18/9 18/13	Any amount, concentration 3x normal serum value lasting for at least 7 days	Ultrasonic dissection Control group	27 31	N/A N/A	N/A N/A	3,7% 25,8%	0,02

Bassi et al. [37]	1999	SC	N/A	N/A	More than 10 mL/d with amylase 1000 IU/L beyond POD 7	Suture closure	15	0%	N/A	33,3%	NS
			N/A	N/A		Suture closure + fibrin glue	11	0%	N/A	27,3%	
			N/A	N/A		Suture closure + polypropylene mesh	15	0%	N/A	13,3%	
			N/A	N/A		Pancreaticojejunostomy	14	0%	N/A	7,1%	
			N/A	N/A		Stapler closure	14	0%	N/A	14,3%	

Suc et al. [38]	2003	MC	N/A	N/A	Any amount, concentration 4x normal serum value lasting for at least 3 days	Occlusion of the main duct with fibrin glue	22	0%	27,20%	18,2%	NS
			N/A	N/A		Control group	19	5,3%	26,30%	15,8%	

Oláh et al. [39]	2009	SC	65 (52–70)** 51 (42–59)**	21/14 20/15	ISGPF	Stapler + seromuscular patch Stapler	35 35	0% 2,8%	11,4%^+^ 31,4%^+^	8,6% 20,0%	N/A

Diener et al. [27]	2011	MC	59,8 ± 14,1* 59,8 ± 13,6*	85/92 76/99	ISGPF	Stapler Suture	177 175	<1% 1%	49,2% 40,0%	35,6% 36,6%	0,84

Frozanpor et al. [24]	2012	SC	N/A N/A	12/15 14/12	ISGPF	Distal pancreatectomy Distal pancreatectomy + stent	27 26	0% 0%	100,0% 100,0%	37,0% 50,0%	0,122

Hamilton et al. [40]	2012	SC	58,6 ± 13,4* 57,5 ± 15,6*	25/21 20/34	More than 50 mL/d, concentration 3x normal serum value after day 3	Stapler Stapler + mesh reinforcement	46 54	0% 0%	60,9% 38,9%	56,5% 38,9%	0,001

Montorsi et al. [41]	2012	MC	60,5 ± 14,9* 61,6 ± 14,2*	64/81 39/91	ISGPF	TachoSil control group	145 130	0% 0%	28,3%^++^ 29,2%^++^	62,1% 68,5%	0,267

Carter et al. [21]	2013	DC	62,5 (29–84)*** 65,0 (20–82)***	22/28 19/32	ISGPF	Falciform patch and fibrin glue control group	50 51	0% 0%	N/A N/A	20,0% 20,0%	1

SC: single center, MC: multicenter, DC: dual-center, N/A: not available, NS: not significant, *mean ± standard deviation, **median (interquartile range), ***median (range), ^+^pancreas-related morbidity, ^++^postoperative complications excluding POPF.

**Table 2:** Assessment of methodological quality and risk of bias of the selected trials.

Reference	Year	Group size calculation	Randomization and concealment of allocation	Blinding	Complete followup	Risk of bias
Suzuki et al. [35]	1995	Missing	Drawing lots	Missing	Missing	High

Suzuki et al. [36]	1999	Missing	Drawing lots	Missing	Missing	High

Bassi et al. [37]	1999	Missing	Missing	Missing	Missing	High

Suc et al. [38]	2003	POPF rate 40%, reduction to 20%, one-tailed test alfa 5%, power of 80%	Telephone call to the coordinating center, computerized random-number tables	Patients and nursing staff	30 days after discharge	Unclear

Oláh et al. [39]	2009	POPF rate 25%, reduction to 15%, alfa 5%, power of 80%	Sealed envelopes	Missing	Hospital stay	Low

Diener et al. [27]	2011	POPF rate 35%, reduction of 15%, two-sided alfa 5%, power of 80%	Central randomisation system	The patient and the outcome assessor	POD 30	Low

Frozanpor et al. [24]	2012	POPF rate 40%, reduction to 0%, two-sided alfa 5%, power of 80%	Opaque sealed envelopes	Missing	POD 30	Low

Hamilton et al. [40]	2012	POPF rate 20%, reduction to 5%, two-sided alfa 5%, power of 80%	Random number generator	The patient and the outcome assessor	POD 30	Low

Montorsi et al. [41]	2012	POPF rate 30%, reduction to 15%, two-sided alfa 5%, power of 80%	Two separate randomization lists at each center (laparoscopic and open)	Missing	2 months after discharge	Low

Carter et al. [21]	2013	POPF rate 30%, reduction to 15%, one-tailed test alfa 5%, power of 80%	Opaque sealed envelopes	Missing	May 2012 (7 months after trial closure)	Low

POD: postoperative day; POPF: postoperative pancreatic fistula.

**Table 3:** Ongoing trials on surgical techniques in distal pancreatectomy.

Department, Country	Study number	Commencement	Planned sample size	Intervention
University of Heildelberg, Heildeberg, Germany [44]	DRKS00000546	December 2010	150	Coverage with falciform ligament versus standard technique

Mayo Clinic, Rochester, MN, USA	NCT01051856	December 2009	400	Stapler closure with bioabsorbable staple line reinforcement (SEAMGUARD) versus radiofrequency ablation device (Tissuelink)

Seoul National University Hospital, Seoul, Republic of Korea	NCT01550406	November 2011	150	TachoComb (collagen sheet coated with fibrinogen) versus polyglycolic acid (biodegradable, thermoplastic polymer)

Wakayama University, Wakayama, Japan	NCT01384617	June 2011	136	Roux-en-Y anastomosis versus stapler closure

Massachusetts General Hospital, Massachusetts, USA	NCT00671463	April 2008	Withdrawn	Placing a stent into the pancreatic duct prior to surgery

## Appendix

### Literature Search

*PubMed: *((((((distal pancreatectomy) AND postoperative complication*)) OR ((“Pancreatectomy” [Mesh]) AND ((“Postoperative Complications” [Mesh]) AND “Pancreatic Fistula” [Mesh]))))) OR (postoperative complication AND pancreatic fistula AND pancreatic resection AND distal pancreatectomy AND pancreas∗).

*Web of Science: *Topic: (distal pancreatectomy) AND Topic: (postoperative complications).

*Embase: *(distal pancreatectomy and postoperative complications) in all fields.

*EBM Review: The Cochrane Central Register of Controlled Trials:* (pancreatectomy AND postoperative complications) in all fields.
